# TOPPITS: Trial Of Proton Pump Inhibitors in Throat Symptoms. Study protocol for a randomised controlled trial

**DOI:** 10.1186/s13063-016-1267-7

**Published:** 2016-04-01

**Authors:** Gillian Watson, James O’Hara, Paul Carding, Jan Lecouturier, Deborah Stocken, Tony Fouweather, Janet Wilson

**Affiliations:** Newcastle Clinical Trials Unit, Faculty of Medical Sciences, Newcastle upon Tyne, NE2 4AE UK; Ear, Nose and Throat Department, The Newcastle upon Tyne Hospitals NHS Foundation Trust, Freeman Hospital, Newcastle upon Tyne, NE7 7DN UK; School of Allied and Public Health, Faculty of Health Sciences, Australian National Catholic University, Brisbane Campus, Queensland, 4014 Australia; Institute of Health and Society, Newcastle University, Newcastle upon Tyne, NE2 4AZ UK

**Keywords:** Ear, nose and throat, Extra oesophageal reflux, Gastro-oesophageal reflux disease, Lansoprazole, Laryngopharyngeal reflux, Otolaryngology, Proton pump inhibitor, Reflux Symptom Index, Throat, Voice

## Abstract

**Background:**

Persistent throat symptoms and Extra Oesophageal Reflux (EOR) are among the commonest reasons for attendance at a secondary care throat or voice clinic. There is a growing trend to treat throat symptom patients with proton pump inhibitors (PPIs) to suppress stomach acid, but most controlled studies fail to demonstrate a significant benefit of PPI over placebo. In addition, patient views on PPI use vary widely.

**Methods/design:**

A UK multi-centre, randomised, controlled trial for adults with persistent throat symptoms to compare the effectiveness of treatment with the proton pump inhibitor (PPI) lansoprazole versus placebo.

The trial includes a six-month internal pilot, during which three sites will recruit 30 participants in total, to assess the practicality of the trial and assess the study procedures and willingness of the patient population to participate. If the pilot is successful, three additional sites will be opened to recruitment, and a further 302 participants recruited across the six main trial sites. Further trial sites may be opened, as necessary. The main trial will continue for a further 18 months. Participants will be followed up for 12 months from randomisation, throughout which both primary and secondary outcome data will be collected. The primary outcome is change in Reflux Symptom Index (RSI) score, the ‘area standard’ for this type of assessment, after 16 weeks (four months) of treatment. Secondary outcomes are RSI changes at 12 months after randomisation, Quality of Life assessment at four and 12 months, laryngeal mucosal changes, assessments of compliance and side effects, and patient-reported satisfaction.

**Discussion:**

TOPPITS is designed to evaluate the relative effectiveness of treatment with a proton pump inhibitor versus placebo in patients with persistent throat symptoms. This will provide valuable information to clinicians and GPs regarding the treatment and management of care for these patients, on changes in symptoms, and in Quality of Life, over time.

**Trial registration:**

ISRCTN38578686. Registered 17 April 2014.

**Electronic supplementary material:**

The online version of this article (doi:10.1186/s13063-016-1267-7) contains supplementary material, which is available to authorized users.

## Background

Trial Of Proton Pump Inhibitors in Throat Symptoms (TOPPITS) addresses the problem of adults with persistent throat symptoms such as globus, catarrh, throat discomfort, clearing, recurring dysphonia or excess mucus. In one UK survey, 6 % of the middle-aged female population had had a persistent feeling of the something in the throat (globus) in the previous 3 months [1]. Globus is also reported to account for up to 4 % of ear, nose and throat (ENT) referrals to secondary care [2]. Throat clearing is the commonest single symptom in any voice clinic. Equally familiar are intermittent hoarse voice and postnasal drip [3]. It is claimed that 55 % of patients referred to a voice clinic have symptoms of extra oesophageal reflux (EOR), while an English study of primary care attenders indicated that 25 % had recent experience of persistent upper respiratory symptoms [4]. In the general population, the lifetime incidence of milder variants of globus (a feeling of a lump in the throat) is over 40 % [5]. In 2010–2011, the Hospital Episode Statistics online database of National Health Service (NHS) activity lists 1,142,404 first ENT consultations. A conservative estimate is that 5 % of these patients were referred for very common throat symptoms like throat clearing, fluctuating voice change, catarrh and chronic throat discomfort, which equates to over 57,000 NHS patients referred to secondary care that year in England alone. Some patients experience anxiety as they fear they may have throat cancer. Even if they have no features and no risk factors for cancer, they may be referred for urgent ENT clinic assessment—a process which prolongs the anxiety, and at times the symptoms. In the absence of good quality treatment algorithms, patients also undergo invasive and costly assessments such as rigid endoscopic examination of the upper aerodigestive tract under general anaesthesia, which typically reveals no significant abnormality, and empiric trials of acid suppression, typically with proton pump inhibitors (PPIs).

EOR symptoms have long been recognised as having a strong placebo response [6]. The original evidence that reflux might affect the upper airway came from animal experiments, and the term ‘acid laryngitis’ was coined 40 years ago [7]. Intracellular reactivation of acidified pepsin may explain pepsin activity at weakly acid pH [8, 9]. There is a growing trend to treat throat symptom patients empirically with PPIs, but most controlled studies fail to demonstrate a significant benefit of PPI over placebo [10, 11, 12]. Our UK Evidence Based Medicine EOR conference concluded that: ‘Studies assessing PPIs in EOR suffer from variable study design and quality, small numbers and heavy selection bias and use a variety of different treatment regimes. The small proportion of controlled studies demonstrating overall benefit of PPI over placebo [13] may incorporate a disproportionate improvement in heartburn, known to respond promptly to antacid therapies, without due regard to the upper airway symptoms per se’ [14, 15]. There was little evidence on other pharmaceuticals such as H2 antagonists [16, 17]. In the Patient and Public Involvement background work for this proposal, individual interviews were conducted with several patients encompassing both young professionals and retired patients. All interviewees fully supported the research proposal. It was also clear, even from a small sample, that patient views on PPIs vary widely but all of them had been treated at some point with PPIs, sometimes on more than one occasion.

## Methods and design

### Aim

This is a non-commercial study to determine the clinical effectiveness of the PPI lansoprazole compared with placebo, in patients referred to secondary care with persistent throat symptoms.

### Objectives

The primary objective is to compare the symptomatic response in patients with persistent throat symptoms at the end of 4 months (16 weeks) of treatment with lansoprazole versus placebo.

Secondary objectives are as follows:

1. To explore the feasibility of study recruitment by means of an internal pilot trial rehearsal whose data will be included in the main data for analysis.
2. To compare the symptom response at the end of 4 months (16 weeks) of treatment with those at 12 months.
3. To determine the utility of the Reflux Symptom Index (RSI) questionnaire, the Comprehensive Reflux Symptom Score (CReSS) questionnaire items and subscales, endolaryngeal examination findings as scored by the Reflux Finding Score (RFS) and patient demographics including age, gender, smoking and body mass index [18–20] as potential baseline determinants of treatment response. (Hence generating improved characterisation of the subgroup of suspected laryngopharyngeal reflux (LPR) patients most likely to benefit from acid suppression therapy.)
4. To compare the patient-reported side effects and compliance with treatment and use of any other over-the-counter medication use in both arms.
5. To compare changes in disease-specific quality of life—Laryngopharyngeal Reflux Health-related Quality of Life (LPR-HRQL)—in the two arms.

### Study setting

Participants for the internal pilot will be recruited from three UK hospitals: The Freeman Hospital in Newcastle upon Tyne (The Newcastle upon Tyne Hospitals NHS Foundation Trust), Sunderland Royal Hospital (City Hospitals Sunderland NHS Foundation Trust) and Queen’s Medical Centre in Nottingham (Nottingham University Hospitals NHS Trust).

On completion of the internal pilot, it is planned that a further three UK sites will participate in the main trial: Royal Sussex County Hospital in Brighton (Brighton and Sussex University Hospitals NHS Trust), Glasgow Royal Infirmary (NHS Greater Glasgow and Clyde) and Manchester Royal Infirmary (Central Manchester University Hospitals NHS Foundation Trust). A further site has also opened: New Queen Elizabeth Hospital, Birmingham (University Hospitals Birmingham NHS Foundation Trust)

If necessary, further sites will be activated as trial recruiting centres.

### Target population and sample size

Participants will be adult patients newly referred to secondary care otolaryngology clinics with persistent (for over 6 weeks) unexplained throat symptoms, principally dysphonia, throat pain, globus sensation (feeling of something stuck in the throat), throat clearing, postnasal drip or mucus excess, and also night-time unexplained cough or choking.

### Inclusion criteria

- Referred with a persistent (over 6 weeks) primary throat symptom—globus, hoarseness, throat clearing, throat discomfort, choking spasms, excess mucus/postnasal drip or otherwise unexplained night-time cough or choking. Score of 10 or more on the non-heartburn items of the RSI.
- Patient has the capacity to provide fully informed consent to participate in the study.

### Exclusion criteria

- Those patients with an RSI score excluding the lower gastrointestinal (GI) item of <10.
- Patients who are not willing to undergo flexible endoscopy could not by definition be included.
- Inability to complete the relevant questionnaires.
- Patients younger than 18 years old.
- Endoscopic evidence of specific laryngopharyngeal pathology that would ordinarily be treated by surgical intervention or be investigated by specific investigations. This includes suspected neoplasia/dysplasia, prominent Reinke’s oedema or unilateral vocal fold polyp and vocal cord palsy, and rarities such as amyloid, Wegener’s and sarcoid granuloma.
- Confirmed or likely, current or prior malignancy of the head and neck or oesophagus.
- Performing voice users including singers, actors and media workers (e.g. voice-over artists, radio DJs).
- Pregnant or lactating woman. Woman of child-bearing potential must be using adequate contraception.
- Currently on acid suppressants, acid neutralisers and alginates and unwilling to discontinue for a 4-week pre-study washout period (PPI); 24 hours for alginate or acid neutraliser. For those discontinuing PPI, ad hoc alginate use is allowed until the final 24–48 hours of the washout period prior to reassessment.
- Prior adverse reaction to PPI.
- Severe hepatic dysfunction.
- Patients taking clopidogrel or warfarin.
- Patients taking phenytoin.
- Patients taking systemic antifungal treatment (specifically itraconazole, ketoconazole, posaconazole, voriconazole).
- HIV-positive patients/patients taking antiviral medications (atazanavir, nelfinavir, raltegravir, saquinavir, tipranavir).
- Patients taking digoxin, cyclosporine, methotrexate, erlotinib, lapatinib, tacrolimus, sucralfate, escitalopram, fluvoxamine, St John’s wort, clozapine, ulipristal or cilostazol.
- Previous participation in this study.
- Use of other investigational study drugs within 30 days prior to study entry.

### Screening, recruitment and consent

#### Identification and screening of participants

Potential participants may be identified through routine clinic outpatient appointments by their treating physician (the principal investigator (PI) at site, or a colleague). The PI and colleagues will ensure that all clinicians at each site are informed about the nature and purpose of TOPPITS, to enhance recruitment.

At the initial clinic appointment a routine consultation and clinical examination will take place. Any patient with persistent throat symptoms will be asked to complete the RSI form, and the total minus the heartburn item will be computed. If a patient scores 10 or more non-heartburn items the physician will inform the patient of the trial and offer them participation.

If a patient scores 10 or more on the RSI—Heart Burn, and is interested in the TOPPITS concept, a participant information sheet (PIS) will be given with an opportunity to view a DVD explaining study procedures (see Additional file 1). The potential participant will be invited to attend the dedicated TOPPITS clinic at a subsequent date convenient for them. The details of such patients will be passed to the TOPPITS research nurse to arrange a convenient appointment. A screening log will be produced which will be filled in for all potential participants screened, including reason for ineligibility and/or refusal to participate.

Potential participants may also be screened using their primary care referral letters. The PIs at site will be responsible for sending out an invitation letter and PIS in the post, detailing the study. These patients will be booked straight onto a TOPPITS trial clinic.

### Recruitment

Eligible patients who have received the PIS through the routine clinic outpatient appointment or through the invite process will then attend a dedicated TOPPITS trial clinic, where any outstanding queries will be answered, followed by an invitation to participate from the consultant or research nurse. They will have all already have received the PIS and a study DVD. They will be given time to discuss the study further in this clinic. At the first trial clinic appointment, a more detailed confirmatory eligibility screen will be completed by the investigator to document participants’ fulfilment of the entry criteria for all patients considered for the study and subsequently included or excluded.

A screening log will be kept to document details of subjects who attend the trial clinic. For subjects who decline participation, the log will record any reasons specified for non-participation.

Patients taking acid suppressants, acid neutralisers or alginates prior to involvement in/being approached to take part in the TOPPITS trial must undergo a 4-week washout period of PPIs or 24 hours for alginates or acid neutralisers, before they start taking TOPPITS trial medication (prescribed as part of Clinic Visit 1). Consent should be taken prior to the washout period. Patients will be provided with a ‘washout period card’ confirming when the washout period started, in case they attend a general practitioner (GP) or clinic appointment prior to Visit 1. This card can then be shown to the GP/consultant to try and ensure the patient does not take/is not prescribed acid suppressants, acid neutralisers or alginates.

#### Consent

Informed consent discussions will be undertaken by appropriate site staff (as per delegation log) involved in the study, including medical staff and research nurses, with the opportunity for participants to ask any questions. Because patients will have already received the patient information at their initial consultation (usually with a different physician to that running the trial clinic) or through the post with an invitation letter, and have taken the positive step to attend the trial clinic, formal TOPPITS consent can be obtained at the first dedicated trial clinic appointment. Those wishing to take part will provide written informed consent by signing and dating the study consent form, which will be witnessed and dated by a member of the research team with documented, delegated responsibility so to do. Written informed consent should always be obtained prior to randomisation and prior to study specific procedures/investigations. Those wishing to have further time to consider may attend a subsequent clinic if they decide to participate.

The original signed consent form will be retained in the Investigator Site File (ISF), with a copy in the clinical notes and a copy provided to the participant. The participant will specifically consent to their GP being informed of their participation in the study.

The right to refuse to participate without giving reasons must be respected.

Because of the small subject population, the patient information sheet, consent form and questionnaires for the study will be available only in English. Interpreters may be arranged for all visits of patients who require them either for verbal translation or for hearing-impaired subjects wishing to take part in the study, via local NHS arrangements. As per local custom, qualified interpreters may be used to explain the consent form and information sheet, and finding the most direct communication will be a priority.

#### Outcomes

The RSI remains the ‘area standard’, and despite well-rehearsed limitations [21] is therefore our chosen primary outcome in the present proposal. Some reported studies have a baseline RSI only just above the normal level, others considerably higher. A Korean observational study of 455 patients reported that the mean RSI score fell from 15 at baseline to <6 after 12 weeks of the PPI rabeprazole [22]. Baseline RSI scores in a much smaller but comparative study of 62 patients treated with esomeprazole were considerably higher (>20) [23], whereas baseline scores in a rabeprazole randomised controlled trial were around 14 [13]. Two trials showed a benefit from a 3-month trial of acid suppression – but Lam et al. [13] continued follow up for a further 6 weeks, when the effect disappeared, while Reichel et al.’s [24] final measurement point was the end of therapy.

Throat symptoms impact on general health status measures [25, 26]. A tool specifically designed to assess throat symptom patients is the LPR-HRQL tool [27, 28], which has also been validated in a Swedish population [29]. Its 43 items are grouped into four domains and an overall impact category, and the tool appears responsive to change [30].

The TOPPITS primary outcome is the change in RSI at 4 months in the treatment and placebo groups in an intention-to-treat analysis. The nine-item RSI total score (0–45) allows comparison with previous studies. We plan, however, to report also the RSI score omitting the heartburn item (0–40), which we and others note can skew the results in favour of PPIs in past small trials. Our proposed analysis will also address the issue of severity variation, through our stratification variables of site and baseline severity.

Secondary outcomes are as follows:

- RSI changes as above, at 12 months after randomisation.
- Quality of life: change in LPR-HRQL total score and subscale at 4 and 12 months.
- Laryngeal mucosal changes—increased RFS—are widely prevalent in both normal volunteers (64–86 %) [31] and in throat symptom patients (38–51 %). The best predictor of EOR is pseudosulcus (67–90 % positive predictive value) [32, 33], but this is a rare finding. We propose to assess the RFS scored 0–29 by an independent observer, blind to treatment group as a response predictor, alongside patient characteristics (age, gender, smoking, body mass index) and the total, and the CReSS total and three subscales—oesophageal (14 items), upper airway (eight items) and pharyngeal (seven items).
- Estimates of compliance (pill count), side effects and current and/or planned use of over-the-counter medication.
- Patient-reported satisfaction.

### Success criteria

The following success criteria were set by the funder, to assess continuation of the study after the first 6 months of recruitment:

- Sites in Newcastle, Sunderland and Nottingham are set up and recruiting.
- The study has recruited a minimum of 30 participants.
- Participants who have reached the first review dates have not experienced any unforeseen problems with prescribed medication.

The objectives of the internal pilot, set by the funder, are as follows:

- To test the projected pathway for throat symptom patients, including high-definition photography for RFS scoring.
- To confirm that eligibility criteria are operational by keeping an anonymous database characterising the total population screened.
- To assess the acceptability and workability of the proposed patient identification and recruitment model.
- To quantify numbers of eligible research clinic attenders and, of those, the number agreeing to be randomised.
- To test whether all of the secondary outcome measures (CReSS and LPR-HRQL) are appropriate.
- To design and test the data storage tools and methods.

### Data handling and record keeping

Data collected as part of this study, their quality and retention are the responsibility of the Chief Investigator, Professor Janet Wilson.

Data will be recorded by authorised site staff on electronic case report forms to allow statistical analysis of the study to take place. Data transferred from site to the secure validated database by remote access will be secure and encrypted.

Data will be handled, computerised and stored in accordance with the Data Protection Act 1998. No participant identifiable data will leave the study site and participants will be identified by unique study numbers.

Caldicott approval covers the collection, retention, storage, transfer and use of personal identifiable information in this trial, and all study data will be retained in accordance with local policy and relevant Standard Operating Procedures (SOPs).

### Sample size calculation

The primary outcome measure is the change in the RSI score from baseline to the 4-month assessment. We believe a clinical effect of 0.4 (equivalent to a difference in change of RSI of 3.1 given a standard deviation of 7.7 [34]) is a reasonable target based on prior LPR therapy studies. Further, an effect size of 0.4 is of smaller magnitude than the effect of phonomicrosurgery or speech therapy. A similar effect size is predicted from the more limited amount of published data available on the LPR-HRQL. For a two-sample *t* test, 90 % power and at the 0.05 significance level, allowing for 20 % loss to follow-up, we require 332 patients (166 in each arm of the study) to give 266 (133 in each arm) completing the study. Two other recent reports of LPR drug studies show lower than 7 % dropout rates. Our NHS experience, however, suggests that this is overly optimistic for a trial of this kind. Because the literature is well populated with underpowered low-impact studies, we prefer to err on the side of caution and hence our sample allows for a 20 % attrition rate.

Participant recruitment into the pilot phase will be for a total of 6 months. We estimate that each site will be able to recruit 10 participants per 3-month block once the study is established. However, as research studies usually take some time to become embedded into practice, we have calculated recruitment across the whole trial based on lower recruitment in three sites during the first 6 months. Our predicted recruitment allows for each of the three sites to recruit 30 % (three participants) in the first 3 months, and 70 % (seven participants) in the second 3 months. We aim for those three sites to be recruiting to full target (10 participants per 3 months) thereafter in the main trial, with equivalent run-in for the remaining three sites.

### Study design and duration

This is a multi-centre, phase III, randomised, double-blind, placebo-controlled trial, with internal feasibility pilot, carried out in secondary care. Patients will be randomised into two parallel streams on a 1:1 ratio stratified by site and baseline severity (two groups, on the basis of the RSI score). Following successful demonstration of recruitment in three sites in the internal pilot, a definitive trial will be conducted over 30 months. Patients with persistent throat symptoms will be identified and recruited from ENT clinics.

#### Feasibility pilot

There will be an initial feasibility pilot, for 6 months, during which three sites will recruit 10 participants each. At the end of month 6, a report will be submitted to the funder, National Institute for Health Research (NIHR) Health Technology Assessment (HTA). The criterion for stopping the trial early is the failure to recruit 30 participants in the pilot, demonstrating a lack of feasibility. Trial progress will be overseen by the Data Monitoring and Ethics Committee (DMEC) and Trial Steering Committee (TSC), and if this target is not reached the HTA will not release the full funding.

#### Full trial

A further 302 participants (332 participants in total) will be recruited over seven sites: Newcastle, Sunderland, Nottingham, Brighton, Glasgow, Manchester and Birmingham.

### Intervention

The active intervention is a 16-week (4-month) course of a 30 mg twice daily dose of the PPI lansoprazole.

### Control

Participants in the control arm will receive a 16-week (4-month) course of twice-daily matched placebo capsule.

### Study compliance and withdrawal

Where feasible, visit windows of ±14 days should ensure visit attendance; non-attendance for study visits will prompt follow-up by telephone.

Compliance with study medication will be assessed by checking and recording the remaining number of capsules at the end of the treatment period by a member of the study team. Study drug accountability will be assessed and documented by local pharmacy before being destroyed.

#### Withdrawal of participants

Study drug must be discontinued if:

- the participant decides that he/she no longer wishes to continue, or
- cessation of study drug is recommended by the PI.

Participants have the right to withdraw from the study at any time for any reason, and without giving a reason. The PI also has the right to withdraw participants from the study drug in the event of intervening pregnancy, inter-current illness, adverse events, serious adverse events, suspected unexpected serious adverse reactions, protocol violations, cure, administrative reasons or other reasons. It is understood by all concerned that an excessive rate of withdrawals can render the study uninterpretable; therefore, unnecessary withdrawal of participants should be avoided. Should a participant decide to withdraw from the study, all efforts will be made to report the reason for withdrawal as thoroughly as possible. Should a participant withdraw from the study drug only, efforts will be made to continue to obtain follow-up data, with his/her permission.

Participants who wish to withdraw from study medication will be asked to confirm whether they are still willing to provide the following:

- Study-specific data at follow-up visits at 4 and 12 months.
- ‘End of study data’ as per the visit at 12 months, at the point of withdrawal.
- If participants agree to either of the above, they will be asked to complete a confirmation of withdrawal form to document their decision.

Participants who withdraw from the study will not be replaced.

### Statistical analysis

The primary outcome measure of change in RSI score after 4 months will be analysed using analysis of covariance (ANCOVA) methods in order to compare the change in RSI between the trial arms while adjusting for potential confounders including the stratification variables used at randomisation of site and baseline severity (as defined by the binary RSI cutoff value) as covariates in the analysis. We will also consider adjusting for gender, age, body mass index, baseline laryngeal appearance scores by the RFS and categories of symptoms. Not all of these covariates mentioned will necessarily be included in the final model but will be considered during the model selection process.

Should data be found to be non-normally distributed the use of transformations or non-parametric approaches will be considered, although as ANCOVA is generally robust to departures from normality the aforementioned approach is likely to be followed. More basic exploratory analyses may be undertaken using the two-sample *t* test or non-parametric alternatives.

The analysis of secondary outcomes will follow a broadly similar strategy when considering questionnaire scores or the change in questionnaire scores. The proportion with follow-up RSI scores within versus out with the published normal range will be analysed using a logistic regression approach, once more adjusting for the covariates as already described for the primary outcome.

The primary analysis will be carried out on an intention-to-treat basis; other analysis groups, such as per protocol, may be considered subsequently. Outcome data will be analysed at the end of the study. There are no planned interim analyses and a full statistical analysis plan will be developed prior to the start of analysis. Safety data will not be subject to statistical analysis. Data with missing observations due to loss to follow-up will be examined to determine both its extent and whether it is missing at random or is informative. If data are missing to a sufficient extent, the use of appropriate multiple imputation techniques will be considered.

### Study monitoring

#### Trial Management Group

The trial will be managed by a Trial Management Group (TMG) consisting of key staff members at the Newcastle Clinical Trials Unit, together with selected investigators, and will meet monthly throughout the set up and duration of the study.

#### Trial Steering Committee

A TSC will be established to provide overall supervision of the trial. The TSC will consist of the Chief Investigator (CI), an independent chairperson, an independent clinician, two public members, an independent statistician and observer members of the TMG. The committee will meet prior to the start of the internal pilot, and then annually during recruitment and for the duration of the trial.

#### Data Monitoring and Ethics Committee

An independent DMEC will be convened to undertake independent review and will monitor efficacy and safety endpoints. The committee will consist of an independent chairperson, an independent clinician and an independent statistician, and will meet to discuss and advise on the inclusion of an interim analysis and possible adoption of a formal stopping rule for efficacy or safety. The committee will then meet at the end of the internal pilot and annually throughout the course of the trial.

### Ethical approval and confidentiality

The conduct of this study will be in accordance with the recommendations for physicians involved in research on human subjects adopted by the 18th World Medical Assembly, Helsinki 1964 and later revisions. All members of the research team and the investigators at each of the participating sites will be trained in those aspects of Good Clinical Practice appropriate to their role in the trial.

A favourable ethical opinion was granted by the NRES Committee—North East: Tyne and Wear South (reference number: 13/NE/0336), and a Clinical Trial Authorisation granted by the Medicines and Healthcare Products Regulatory Agency, prior to commencement of the study. Local approvals are sought before recruitment commences at each site. The Newcastle Clinical Trials Unit in its capacity as The Study Co-ordination Centre requires a written copy of local approval documentation before initiating each site and accepting participants into the study.

Information sheets will be provided to all eligible subjects. Eligible subjects may also be given access to a DVD that explains more about the study. Written informed consent will be obtained prior to any study procedures.

#### Safeguarding confidentiality

Personal data will be regarded as strictly confidential. To preserve anonymity, any data leaving the site will identify participants by a unique study identification code only. The study will comply with the Data Protection Act 1998. All study records and ISFs will be kept at site in a locked filing cabinet with restricted access.

#### Long-term data storage

At the end of the study, original questionnaires, case report forms and consent forms will be securely archived for 15 years following publication of the last paper or report from the study, in line with sponsor policy and standard operating procedures. This will allow any queries or concerns about the data, conduct or conclusions of the study to be resolved.

## Discussion

Throat symptoms represent a significant health issue, and there is wide uncertainty on PPI use among clinicians, GPs and patients. In assessing changes in symptoms and in overall quality of life, the TOPPITS trial will provide a clear assessment of their use, in a randomised, placebo-controlled trial, and robust information on their use.

### Trial status

At the time of manuscript submission, 201 participants have been recruited to the trial and it remains open to recruitment.

## Additional files

The following documents were submitted with this protocol for publication: CONSORT 2010 checklist of information to include when reporting a randomised trial (see Additional file 1), and Standard Protocol Items: Recommendations for Interventional Trials (SPIRIT) 2013 Checklist—recommended items to address in a clinical trial protocol and related documents (see Additional file 2). Additional file 1: presents the CONSORT 2010 checklist of information to include when reporting a randomised trial, completed for TOPPITS. (PDF 43 kb) Additional file 2: presents the SPIRIT 2013 Checklist—recommended items to address in a clinical trial protocol and related documents completed for TOPPITS. (PDF 35 kb)

## Abbreviations

ANCOVA: Analysis of covariance
CReSS: Comprehensive Reflux Symptom Score
DMEC: Data Monitoring and Ethics Committee
ENT: Ear, nose and throat
EOR: Extra oesophageal reflux
GP: General practitioner
HTA: Health Technology Assessment
ISF: Investigator Site File
LPR: Laryngopharyngeal reflux
LPR-HRQL: Laryngopharyngeal Reflux Health Related Quality of Life
NHS: National Health Service
NIHR: National Institute for Health Research
PI: Principal investigator
PIS: Participant information sheet
PPI: Proton pump inhibitor
RFS: Reflux Finding Score
RSI: Reflux Symptom Index
TMG: Trial Management Group
TOPPITS: Trial Of Proton Pump Inhibitors in Throat Symptoms
TSC: Trial Steering Committee
